# Evaluation of real-time PCR targeting the *lipL32* gene for diagnosis of *Leptospira* infection

**DOI:** 10.1186/s12866-020-01744-4

**Published:** 2020-03-11

**Authors:** Daša Podgoršek, Eva Ružić-Sabljić, Mateja Logar, Andrea Pavlović, Tatjana Remec, Zvonko Baklan, Emil Pal, Tjaša Cerar

**Affiliations:** 1 Institute of Microbiology and Immunology, Faculty of Medicine, University of Ljubljana, Zaloška 4, SI-1000 Ljubljana, Slovenia; 2 Department of Infectious Diseases, University Medical Centre Ljubljana, Japljeva 2, SI-1000 Ljubljana, Slovenia; 3 Department of Infectious Diseases, General Hospital Celje, Oblakova ulica 5, SI-3000 Celje, Slovenia; 4 Department of Infectious Diseases, General Hospital Novo Mesto, Šmihelska cesta 1, SI-5000 Novo Mesto, Slovenia; 5 Department of Infectious Diseases, University Medical Centre Maribor, Ljubljanska ulica 5, SI-2000 Maribor, Slovenia; 6 Department of Infectious Diseases, General Hospital Murska Sobota, Ulica dr. Vrbnjaka 6, SI-9000 Murska Sobota, Slovenia

**Keywords:** Leptospira, PCR, Lipl32, Leptospirosis

## Abstract

**Background:**

Different diagnostic methods have been used for the laboratory confirmation of leptospirosis. Molecular diagnostic techniques are not only faster and more sensitive than culture analysis, but can also detect a *Leptospira* infection before the appearance of antibodies. The aim of the present study was to analyze and compare two different PCR approaches applied to blood and urine specimens obtained from patients with clinical manifestations that were suggestive of leptospirosis. Furthermore, the results of these different PCR approaches were compared with the results of culture and serology analyses.

**Results:**

A total of 400 samples (234 blood or 58.5% and 166 urine of 41.5%) from 310 Slovenian patients with clinical manifestations suggestive of leptospirosis were tested using conventional PCR assays targeting the *rrs* gene and RT-PCR targeting the *lipL32* gene. Additionally, culture, serology and sequence analysis were performed for the majority of these samples. The PCR and RT-PCR results were concordant in 376 out of 400 of these samples (94.0%). Conventional PCR was positive for 27 out of 400 samples (6.8%) and RT-PCR was positive for 47 out of 400 samples (11.8%). Culture and microscopic agglutination tests supported these diagnoses.

**Conclusions:**

A comparison of the two PCR methods indicated that the RT-PCR targeting of the *lipL32* gene was faster, more sensitive and more specific for the determination of *Leptospira* DNA in these clinical samples.

## Background

Leptospirosis is an acute febrile zoonotic disease that is caused by spirochetes bacteria of the genus *Leptospira*. This disease is prevalent in tropical and subtropical regions, and has been reported in other parts of the world [1, 2]. In Slovenia, leptospirosis is endemic in the most eastern region of the country, known as Pomurje. Indeed, up to 30 cases per year of *Leptospira* infection are reported in Slovenia [3].

Rodents constitute the main carrier of *Leptospira*, although many other free-living and domesticated animals can also become infected. These bacteria are excreted in the urine of infected animals, and can thus contaminate fields, meadows, and standing and running water. *Leptospira* infection in humans can arise from direct contact with an infected animal or from indirect contact with a contaminated environment. In Slovenia, the risk of acquiring leptospirosis is associated with occupational and recreational exposure [3].

Leptospirosis can seen in forms ranging from mild influenza-like symptoms to severe jaundice, renal failure and bleeding, and can result in the death of the patient [1, 4]. The clinical diagnosis of *Leptospira* infection can also be confused with other febrile illnesses due to the similarity of clinical symptoms [1, 3–5]. The early diagnosis of *Leptospira* and subsequent antimicrobial therapy are thus very important for both clinicians and patients in order to reduce patient mortality and morbidity.

Different diagnostic methods have been described for the confirmation of leptospirosis [1, 4, 6]. Serological tests based on the presence of specific antibodies against *Leptospira* are typically used in routine diagnosis. The main problem here is that the antibodies became detectable from one to 2 weeks after clinical presentation, or even later, which is too late for early antibiotic treatment. Instead, the microscopic agglutination test (MAT) is considered the reference test for leptospirosis [4, 6]. The culturing of samples is also a reliable method, but is time consuming and has low sensitivity [1, 6, 7]. In contrast, molecular diagnostic techniques (e.g., polymerase chain reaction [PCR]) are faster and appear to be more sensitive than culturing, and can detect *Leptospira* directly in specimens [4, 8, 9]. Thus, PCR can confirm infection earlier than serological tests. More recently, the development of real-time (RT-)PCR was a revolution for the molecular diagnosis of infectious diseases. RT-PCR also has several advantages over conventional PCR, as it is easier to perform and less time consuming, shows reduced variability and contamination, facilitates online monitoring, and does not require post-reaction analyses [9, 10].

Several RT-PCR assays that amplify different target sequences have been described for the diagnosis of *Leptospira* infection [4, 8, 9, 11–15]. The majority of these studies have indicated a primary role for the *lipL32* gene, which encodes the *Leptospira* subsurface lipoprotein Lipl32 [16] . Because Lipl32 is believed to be a virulence factor that is only presented in pathogenic species, this provides for the selective detection of the pathogenic *Leptospira* and helps to increase the specificity of these methods [17, 18].

The aim of the present study was to analyze and compare two different PCR approaches applied to blood and urine specimens obtained from patients with clinical manifestations that were suggestive of leptospirosis. Furthermore, the results of these different PCR approaches were compared with the results of culture and serology analyses.

## Results

To detect *Leptospira* DNA in blood and urine samples of patients with clinically suspected leptospirosis, two PCR approaches were used that amplified two different target DNA sequences: the *rrs* and *lipL32* genes. In addition to these molecular tests, culture and MAT were performed on samples from the majority of the patients.

This study included 400 specimens of blood (*n* = 234) and urine (*n* = 166) from 310 patients with suspected *Leptospira* infections. Both blood and urine specimens were received from 66 of the 310 patients, while only one type of sample (i.e. blood or urine) for the rest of the patients (224 our of 310) was referred to the analysis laboratory. Thus, including these together, there were 66 blood–urine paired specimens, and 168 single blood samples and 100 single urine samples, from 310 different patients from different hospitals in Slovenia.

The conventional PCR that targeted the *rrs* gene gave positive result in 27 out of 400 samples (6.8%), while the RT-PCR that targeted the *lipL32* gene was positive in 47 out of 400 samples (11.8%). This difference was statistically significant (*p* = 0.0001). The results from each PCR and comparisons of the two tests are given in Table 1. These results across both tests were consistent in 376 out of 400 cases (94.0%), with 25 samples positive and 351 samples negative with both of these assays (Table 1).

**Table 1:** Results for the detection of leptospiral DNA using conventional PCR and RT-PCR across 400 samples of either blood or urine

Conventional PCR	RT PCR								
	Blood			Urine			Blood & Urine		
	Pos (%)	Neg (%)	All (%)	Pos (%)	Neg (%)	All	Pos (%)	Neg (%)	All
Pos	16 (6.8)	0	16 (6.8)	9 (5.4)	2 (1.2)	11 (6.6)	25 (6.3)	2 (0.5)	27 (6.8)
Neg	15 (6.4)	203 (86.8)	218 (93.2)	7 (4.2)	148 (89.2)	155 (93.4)	22 (5.5)	351 (87.7)	373 (93.2)
All	31 (13.2)	203 (86,8)	234 (100)	16 (9.6)	150 (90.4)	166 (100)	47 (11.8)	353 (88.2)	400 (100)
p (Chi sq)	0.0003			0.1824			0.0001		
Kappa	0.6492			0.6383			0.6453		

Using the RT-PCR with these blood and urine samples, 31 out of 234 samples (13.2%) and 16 out of 166 samples (9.6%) were positive, respectively, compared to conventional PCR reactions with 16 out of 234 positive samples (6.8%) and 11 out of 166 (6.6%) positive samples, respectively (Table 1). These differences between the positive results for the two PCR protocols reached significance in the case of blood samples (*p* = 0.0003), but not for urine (*p* = 0.1824). These two PCR tests were simultaneously positive in 16 out of 234 (6.8%) cases and 9 out of 166 cases (3.8%), respectively (Table 1).

Table 2 lists all of the patients where positivity was seen for at least one of the two PCR assays, along with results from culture and/or serology analyses. The results of the sequence analysis on the products of some of the positive conventional PCRs are also reported (Table 2).

**Table 2:** Patients (Pt) who were positive according to at least one of the three conventional PCR assays or according to RT-PCR, including results from conventional PCR product sequence analysis, and from culture and serology (i.e. microscopic agglutination test [MAT] on blood) analyses

Pt	Sample	Conventional PCR					RT-PCR	Culture	MAT
		LeptoA/B	L3/4	L4/ LPato2	Final result	Product sequence analysis			
1	blood	pos^a^	neg	pos^a^	pos	^a^*Leptospira interrogans*	pos	neg	Pos 1600 Sejroe
1	urine	pos^a^	neg	pos^a^	pos	^a^*Leptospira* interrogans	pos	neg	
2	urine	pos	neg	pos	pos	NT	pos	neg	neg
3	urine	pos	neg	pos	pos	NT	pos	pos	Pos 1600 Icterohaemorrhaiae
4	urine	pos	neg	pos	pos	NT	pos	neg	Pos 600 Australis
5	blood	neg	neg	neg	neg	NT	pos	neg	Pos 25,600 Cynopteri
6	blood	pos	neg	pos	pos	NT	pos	neg	neg
7	blood	pos	neg	pos	pos	NT	pos	neg	Pos 100 Australis
8	blood	neg	neg	neg	neg	NT	pos	neg	Pos 102,400 Tarassovi
9	blood	neg	neg	neg	neg	NT	pos	neg	neg
10	urine	pos^a^	neg	pos^a^	pos	^a^*Leptospira kirschneri*	neg	neg	neg
11	blood	neg	neg	neg	neg	NT	pos	neg	Pos 1600 Sejroe
12	blood	pos^a^	neg	pos^a^	pos	^a^*Leptospira* spp.	pos	neg	Neg
13	urine	neg	neg	pos^a^	neg	^a^*Leptospira* spp.	pos	NT	Neg
14	blood	neg	neg	neg	neg	NT	pos	neg	Pos 800 Australis
15	blood	neg	neg	pos^a^	neg	^a^*Leptospira interogans*	pos	pos	Pos 200 Canicola
16	blood	pos^a^	neg	pos^a^	pos	^a^*Leptospira* spp.	pos	neg	neg
16	urine	pos^a^	neg	pos^a^	pos	^a^*Leptospira* spp.	pos	neg	neg
17	blood	neg	neg	pos^a^	neg	^a^*Leptospira* spp.	pos	pos	NT
18	blood	pos	neg	pos	pos	NT	pos	pos	Pos 400 Grippotyphosa
19	blood	pos	neg	pos	pos	NT	pos	NT	neg
20	blood	neg	neg	neg	neg	NT	pos	neg	Pos 200 Australis
20	urine	neg	neg	neg	neg	NT	pos	neg	
21	blood	neg	neg	neg	neg	NT	pos	NT	NT
22	blood	neg	neg	pos^a^	neg	^a^*Leptospira* spp.	pos	NT	NT
23	blood	pos	neg	pos	pos	NT	pos	NT	neg
23	urine	pos	neg	pos	pos	NT	pos	NT	
24	urine	pos^a^	neg	pos^a^	pos	^a^*Leptospira meyeri*	pos	pos	Pos 400 Sejroe
24	blood	pos^a^	neg	pos^a^	pos	^a^*Leptospira meyeri*	pos	pos	
25	blood	neg	neg	pos^a^	neg	^a^*Leptospira* spp.	pos	NT	Pos 200 Grippotyphosa
26	blood	neg	neg	pos^a^	neg	^a^*Leptospira* spp.	pos	neg	Pos 200 Australis
27	blood	pos^a^	neg	pos^a^	pos	^a^*Leptospira* spp.	pos	neg	neg
28	urine	neg	neg	neg	neg	NT	pos	neg	neg
29	blood	neg	neg	neg	neg	NT	pos	neg	Pos 3200 Copenhageni
30	blood	neg	neg	neg	neg	NT	pos	pos	neg
31	urine	neg	neg	neg	neg	NT	pos	neg	Pos 400 Panama
32	blood	neg	neg	neg	neg	NT	pos	pos	NT
33	blood	pos^a^	neg	pos^a^	pos	^a^*Leptospira interogans*	pos	pos	neg
34	blood	pos	neg	pos	pos	NT	pos	neg	neg
34	urine	pos	neg	pos	pos	NT	pos	neg	
35	blood	pos	neg	pos	pos	NT	pos	neg	neg
36	blood	pos^a^	neg	pos^a^	pos	^a^*Leptospira* spp.	pos	neg	neg
37	urine	neg	neg	pos^a^	neg	^a^*Leptospira* spp.	pos	neg	Pos 3200 Sejroe
38	urine	pos	neg	pos	pos	NT	pos	neg	Pos 800 Grippotyphosa
39	blood	pos	neg	pos	pos	NT	pos	pos	Pos 1600 Icterohaemorrhaiae
40	urine	neg	neg	neg	neg	NT	pos	neg	Pos 200 Australis
41	blood	pos	neg	pos	pos	NT	pos	neg	neg
41	urine	neg	neg	neg	neg	NT	pos	neg	
42	urine	pos^a^	neg	pos^a^	pos	^a^*Atopobium vaginae*	neg	neg	neg

*NT* not tested

^a^ Conventional PCR products subjected to sequence analysis

Among 66 of the 310 patients who provided paired blood–urine samples for PCR testing, five patients were blood and urine positive for *Leptospira* according to both PCR protocols (Table 2; patients #1, #16, #23, #24 and #34) and one patient was only blood and urine positive according to the RT-PCR (Table 2, patient #20), while one patient was blood positive according to both tests, but only showed urine positivity according to the RT-PCR (Table 2; patient #41). In four of these 66 patients, *Leptospira* infection was also confirmed using other diagnostic methods (i.e. culture and MAT) and/or by the sequence analysis of conventional PCR products (Table 2; patients #1, #16, #20 and #24). A large majority of the patients whose paired blood–urine samples were simultaneously sent to the laboratory were PCR negative for both of these PCR protocols (59 out of 66).

It is evident from Table 1 that 25 samples were positive for both PCR protocols, and that these samples belonged to 20 out of 310 patients. Six of these patients with paired blood–urine samples are mentioned above (Table 2; patients #1, #16, #23, #24, #34 and #41, with the exception of a urine sample). In the remaining 14 of these patients, leptospirosis was confirmed using additional diagnostic assays. Of these 10 patients, three were culture- and MAT-positive (Table 2; patients #3, #18 and #39), one was culture positive, but MAT-negative (Table 2; patient #33) and three were MAT-positive, but culture negative (Table 2; patients #4, #7 and #38). The sequence analysis of the conventional PCR amplicons of three of the culture and MAT-negative patients showed evidence of *Leptospira* DNA (Table 2; patients #12, #27 and #36).

Table 1 also shows that 22 out of 400 samples (5.5%) from 21 patients were negative according to conventional PCR, but positive according to RT-PCR. Three of these samples belonged to two patients, who are mentioned above (Table 2; patients #20 and #41). Culture, MAT and sequence analyses were performed to confirm these as *Leptospira* infections for the majority of the remaining 19 patients. Leptospirosis was confirmed in 16 of these 19 patients: one out of 16 was culture- and MAT-positive (Table 2; patient #15), three out of 16 were culture positive (Table 2; patients #17, #30 and #32) and 10 out of 16 patients were MAT-positive (Table 2; patients #5, #8, #11, #14, #25, #26, #29, #31, #37 and #40). The PCR products were sequenced for two out of 16 patients where culture and MAT analyses were negative or were not performed (Table 2; patients #13 and #22), while a nucleotide sequence analysis showed the presence of *Leptospira* in both cases. In three out of 19 patients (Table 2; patients #9, #21 and #28), *Leptospira* infection was not confirmed using other diagnostic methods, or other diagnostic methods were not applied.

On the other hand, two out of 310 patients (0.7%) were positive according to conventional PCR and negative using RT-PCR (Table 3), as samples that originated from urine. Both of these patients were culture- and MAT-negative (Table 2; patients #10 and #42). The sequence analysis showed identity to *Atopobium vaginae* for one of these samples, which suggests false positive results with conventional PCR, while *Leptospira* was confirmed in the other sample.

**Table 3:** Primers and probe sequences for polymerase chain reactions (PCR) performed in the study

Primer	Sequence
L3	5′ – TgA ggg TTA AAA CCC CCA AC – 3`
L4	5′ – gAT TTT TCg ggT AAA gAT T – 3`
LeptoA	5′ – ggC gCg TCT TAA ACA Tg – 3`
LeptoB	5′ – TTC CCC CCA TTg AgC AAg ATT – 3`
LPato2	5′ – TCA CAT Y gCT TAT TTT – 3`
LipL32-45F	5′ – AAg CAT TAC CgC TTg Tgg Tg – 3’
LipL32-286R	5′ – gAACTCCCATTTCAgCgATT – 3’
Probe LipL32-189P	5′ – LC610-AAA gCC Agg ACA AgC gCC g--BHQ2–3’

As indicated, a sequence analysis was performed on PCR amplicons obtained from the conventional PCR approach (as three reactions per set). *Leptospira* DNA was confirmed in 11 samples from eight patients whose final result was positive (Table 2; patients #1, #10, #12, #16, #24, #27, #33 and #36), and in seven samples from seven patients whose final result was negative (Table 2; patients #13, #15, #17, #22, #25, #26 and #37). Moreover, there was a sample with a positive final result using conventional PCR where the sequence analysis did not confirm the presence of *Leptospira* DNA (Table 2, patient #42).

Taken together, the RT-PCR that was based on amplification of the *lipL32* gene was significantly more sensitive than the traditional PCR that was based on amplification of the *rrs* gene (*p* = 0.0001). Culture analyses were performed for 34 out of 42 patients who were positive according to at least one of the two PCR assays (Table 2), where 8 out of 42 patients (19.1%) were culture positive. It is evident from Table 2 that culture analyses were always simultaneously positive with the other microbiological tests performed. MAT analyses were performed for 38 out of 42 patients who were positive according to at least one of the two PCR protocols (Table 2), with 20 out of 42 of these patients (47.6%) being MAT-positive. It can be seen from Table 2 that MAT analyses were simultaneously positive with RT-PCR for a larger majority of patients.

## Discussion

Suspicion of leptospirosis is based on epidemiological data, and patient clinical symptoms and signs. As the clinical characteristics of leptospirosis can be confused with other infections, this highlights the great importance of the laboratory diagnosis of these infections [1, 4, 5]. Due to the low sensitivity of culture analyses and the delayed appearance of antibodies (i.e. 2 weeks or more after the onset of symptoms), molecular approaches are welcome for the early microbiological diagnosis of *Leptospira* infection [4, 6, 7, 9]. The detection of *Leptospira* DNA in the first few days of infection facilitates early antibiotic treatment, and thus promotes successful recovery for these patients [4, 8]. For this reason, molecular diagnosis plays an important role in the treatment of patients with leptospirosis all over the world, including in Slovenia. The present study was designed to compare two different PCR approaches (i.e. conventional PCR and RT-PCR) in patients with clinical manifestations that were suggestive of leptospirosis, and to compare the results of these two PCR approaches with the results of culture and serology analyses.

Different molecular approaches based on different target sequences, molecular methods and study design can be found in literature [4, 8, 9, 11–15]. In recent years, several RT-PCR assays have been described [9, 10]. In the present study, we evaluated a RT-PCR protocol that targets the *lipL32* gene, which has high sequence conservation and is the most copious *Leptospira* protein (i.e. 38,000 copies per cell), thus supporting *lipL32* as an appropriate target sequence [17, 18]. The results of *lipL32* RT-PCR assays were compared with results using conventional PCR according to three conventional PCR reactions (i.e. LeptoA/B, L3/L4, L4/LPato2) that targeted various regions of the *rrs* gene that are highly conserved throughout the bacterial kingdom. This study also included culture analyses and serological MAT tests to confirm leptospirosis.

Comparisons of the results of both of these PCR tests showed higher sensitivity for the RT-PCR, as RT-PCR confirmed leptospiral infection in 22 samples (from 21 patients), while traditional PCR revealed negative results for all of these patients. Using two-tailed McNemar tests, the differences between the results of these tests were statistically significant (*p* = 0.0001).

The specificity of the PCR amplicon in the conventional PCR approach was defined using a sequence analysis. In the present study, the conventional PCR gave one false-positive result when performed on urine samples (i.e. patient #42), while the RT-PCR correctly identified the absence of *Leptospira* DNA in the same urine sample. The explanation for false-positive results might lie in the fact that *rrs* genes in conventional PCR are usually more prone to unexpected cross-reactivity, especially when performed on urine samples, which can contain bacteria that are part of the normal flora of the genital and urinary tract [15].

On the other hand, there was a case with a positive result for conventional PCR that was confirmed as *Leptospira* infection through sequence analysis, but where negative results were obtained for RT-PCR, and culture and serology analyses (patient #10). We believe that the negative result for the RT-PCR was due to the fact that this was performed retrospectively, where the sample had been stored for 4 years. There was thus the possibility of DNA degradation in this sample. The possibility of contamination of the conventional PCR during the analytical procedures can be excluded due to the follow-up working conditions of the technique.

The RT-PCR showed higher rates of positivity when performed on blood samples than for the conventional PCR, which would appear to be because of the lower diagnostic sensitivity of the conventional PCR. This low positivity might be due to low bacteremia in some patients, which can be caused by the presence of specific antibodies in the patient’s blood (i.e. nine out of the 15 blood samples that were RT-PCR-positive and conventional PCR-negative were MAT-positive) or by empiric antibiotic treatment. These factors are generally known to decrease the positive results of DNA amplification methods [1].

According to the findings of the present study, this RT-PCR protocol has several advantages over conventional PCR protocols. In particular, it is faster and facilitates the detection of amplified fragments during the process and therefore does not require end-point detection, which is very time consuming and less precise. Furthermore, there is less chance of contamination, as the entire process from amplification of the target region to analysis of the amplified DNA is performed in one tube.

Culture and serological tests were performed in the majority of these patients. Culture analysis is known to be time consuming and show low sensitivity, although it remains a valuable diagnostic method and is the best proof of infection. Moreover, an isolated *Leptospira* strain can then be typed to identify its serovar, which is of great importance in epidemiological studies. On the other hand, serological tests are the most commonly used diagnostic method for *Leptospira*, although detectable titers of antibodies in the blood usually appear about 5–10 days after the onset of the disease [6]. In the present study, culture analysis was rarely positive, and when it was, this occurred simultaneously with the other tests performed. This was particularly true of the case with RT-PCR, while there were also a number of MAT-positive patients. In the case of inconclusive results of PCR tests, MAT can significantly contribute to the final diagnosis of a disease. The patients in the present study who lacked other microbiological tests were not classified as *Leptospira* infections from a clinical point of view, and no additional samples were sent to the laboratory for serology and/or cultivation.

## Conclusion

We compared two different PCR approaches that were supported by culture, MAT and sequence analysis. The RT-PCR targeting of the *lipL32* gene was shown to be preferred to conventional PCR targeting of the *rrs* gene, as it is quicker, easier to perform, less prone to contamination and more sensitive for the determination of *Leptospira* DNA in these clinical samples. This RT-PCR is also more sensitive than culture analyses and quicker than serological tests [19]. Thus, according to these findings, this RT-PCR protocol represents a useful tool for the rapid microbiological diagnosis of acute leptospirosis.

## Methods

### Patients and samples

Samples of blood (in EDTA) and urine were obtained from patients with suspected leptospirosis, and were used for molecular diagnosis. A suspected diagnosis of leptospirosis was defined as acute febrile illness with headache, myalgia, arthralgia, meningeal irritation and renopatia. Whole blood samples from patients were used for antibody detection. For some patients, whole blood (in blood culture bottles) and urine (in sterile bottles) were collected aseptically for the analysis and isolation of *Leptospira*.

The patients were observed in different hospitals in Slovenia from January 2001 to December 2014. All of the clinical specimens were collected during initial patient examinations and were sent to the Institute of Microbiology and Immunology in Ljubljana (Slovenia). Some of these patients (~ 20%) have already been included in an evaluation of an immune-chromatographic (Leptocheck) test for the detection of specific antibodies against *Leptospira* [20].

### Nucleic acid extraction from blood and urine samples

The blood specimens in EDTA were centrifuged at 200x *g* for 10 min and the plasma was sampled for DNA extraction. Urine specimens were centrifuged at 14100x *g* for 30 min, the supernatant was removed and the pellets were resuspended with 180 μL MagNA Pure Bacteria Lysis Buffer (Roche, Mannheim, Germany) and 20 μL proteinase K (Roche, Mannheim, Germany). These samples were then incubated for ≥10 min at 65 °C, and then for 10 min at 95 °C. The total DNA was then extracted using an automated method on a MagNA Pure Compact apparatus (Roche, Germany), in accordance with the manufacturer’s instructions. The extracted DNA was stored at − 20 °C until testing [21].

### PCR amplification

The samples were analyzed for the presence of *Leptospira* DNA using two different PCR approaches. To avoid PCR contamination and amplicon carry-over, the samples were processed in separate rooms, with the use of plugged pipette tips. A panel of positive and negative control samples was included in each run to monitor amplification and any potential contamination.

The first approach included three different PCR reactions that targeted different parts of the *rrs* gene, with these three PCR protocols indicated here as ‘conventional PCR’. The first of these amplified the DNA of both pathogenic and nonpathogenic *Leptospira* spp. (LeptoA/ LeptoB), while the other two conventional PCR protocols followed one another as nested PCR (L3/L4; L4/Lpato2), with the amplification of DNA of the pathogenic *Leptospira* spp. only. The reactions were performed using the primers and protocols described by Merien et al. [22] and Murgia et al. [23]. The amplified products were analyzed on 1% agarose gels that were stained with ethidium bromide. Under each of the runs for each of the samples, any of these three PCR reactions might be positive, although a final positive result was only considered when the results of the first LeptoA/LeptoB step and the nested L4/Lpato2 analyses were both positive [4, 8].

A further PCR protocol was designed as RT-PCR using primers and a probe that targeted the *lipL32* gene. The amplification was performed using a RT-PCR machine (LightCycler; Roche, Germany), while a data analysis was performed using the detection system of the RT-PCR machine [12, 13].

The primer sequences and reaction conditions for all of these PCR reactions are given in Tables 3 and 4.

**Table 4:** Protocols used for conventional PCR and RT-PCR

Stage	PCR									RT-PCR		
	**LeptoA/LeptoB**			**L3/L4**			**L4/LPato2**					
	**Temp. (°C)**	**Time (min)**	**Cycles (n)**	**Temp. (°C)**	**Time (min)**	**Cycles (n)**	**Temp. (°C)**	**Time (min)**	**Cycles (n)**	**Temp. (°C)**	**Time (s)**	**Cycles (n)**
Initial denaturation	93	3	1	93	3	1	93	3	1		600	
Denaturation	93	1	35	93	1	35	93	1	35	95	15	45
Annealing	60	1		57	1		52	1		53	10	
Elongation	72	1		72	1		72	1		72	10	
Final elongation	72	9	1	72	9	1	72	9	1	40	30	1
Hold	4			4			4					

The analytical sensitivity of the RT-PCR targeting LipL32 gene was determined using 10-fold dilutions of gBlocks (Integrated DNA Technologies Inc., Coralville, IA) fragments ranging from 10^7^ to 10^0^ copies/μL. All dilutions were tested in triplicate, while a negative control was included in the run. The RT-PCR was able to detect 10^7^ to 10^0^ copies/μL, while PCR efficiency was 1.981. Patients’ samples were considered positive if the cycle threshold (Ct) value was less than 37.

### Sequencing

About half of the PCR products from the conventional PCR approach underwent sequence analysis, with the aim of confirming the presence of *Leptospira* DNA in these samples. The PCR products were initially purified (PCR Product Clean-Up; Applied Biosystems, Thermo Fisher Scientific, USA) in accordance with the manufacturer’s instructions. The reaction mixture contained 5 μL of purified PCR product, 0.5 μL of exonuclease I and 1 μL of shrimp alkaline phosphatase. These were incubated at 37 °C for 15 min. The reaction was then stopped by heating to 85 °C for 15 min [24].

For sequencing the purified PCR products, the same primers as for the PCR reactions were used. Each 20 μL reaction mixture contained 5.0 μL of purified PCR product, 8.7 μL of deionized RNase/DNase-free H_2_O, 2.0 μL of Big Dye Terminator v3.1 Cycle Sequencing RR-100 MasterMix (Applied Biosystems, Thermo Fisher Scientific, USA), 3.0 μL of Big Dye Terminator v3.1 5× Sequencing Buffer (Applied Biosystems, Thermo Fisher Scientific, USA) and 1.3 μL of each primer. Cycle sequencing was performed using initial denaturation at 96 °C for 1 min, followed by 25 cycles of 10 s at 96 °C, 5 s at 50 °C and 4 min at 60 °C in a thermal cycler (Veriti; Applied Biosystems, Thermo Fisher Scientific, USA). The sequencing products were purified using purification kits (BigDye XTerminator; Applied Biosystems, Thermo Fisher Scientific, USA) and sequenced on an automated sequencer (ABI-3500; Applied Biosystems, Thermo Fisher Scientific, USA) [25]. The sequences were assembled using the CLC Genomics Workbench 6 (CLC Bio, Qiagen, Germany) and analyzed using Ribosomal Database Project sequence matching (http://rdp.cme.msu.edu/seqmatch/seqmatch_intro.jsp).

### Microscopic agglutination test

The blood for antibody detection was collected by venepuncture during initial patient examinationss and was allowed to clot. After centrifugation at 5000×*g* for 10 min, the sera were collected and stored at − 20 °C until testing. The sera were examined using the MAT, with a panel of 15 serovars: Gryppotyphosa, Canicola, Sejroe, Pomona, Cynopteri, Copenhageni, Patoc, Australis, Autumnalis, Pyrogenes, Bataviae, Tarassovi, Castellanis, Panama and Javanica, as reported to be suitable for the geographic area [6]. The sera to be tested were diluted serially and live *Leptospira* antigen suspensions from a battery of 15 serovars were added. The serum/ *Leptospira* culture mixtures were incubated at 37 °C for 1 h and then examined under dark-field microscopy for *Leptospira* agglutination, and the titers were determined. Titers of ≥100 were deemed positive [4, 6].

### *Leptospira* isolation

Approximately 1 mL of each sample (i.e. whole blood or urine) was inoculated into a tube containing 7 mL of Ellinghausen–McCullough–Johnson–Harris liquid medium, with more than one tube inoculated per sample. The tubes were incubated at 28 °C for 9 weeks and then examined for *Leptospira* growth once a week, using dark-field microscopy [6, 7].

### Statistical analysis

To compare the performance of the two PCR methods, the kappa value and McNemar’s chi-square value were calculated using an Epitools epidemiological calculator [26].

## Data Availability

The data that support the findings of this study are available from the corresponding author (TC) upon reasonable request.

## Abbreviations

MAT: Microscopic agglutination test
RT-PCR: Real-time polymerase chain reaction
